# MiR-31-5p alleviates septic cardiomyopathy by targeting BAP1 to inhibit SLC7A11 deubiquitination and ferroptosis

**DOI:** 10.1186/s12872-024-03954-4

**Published:** 2024-05-30

**Authors:** Yafeng Liu, Niandan Hu, Bo Ai, Hao Xia, Wenqiang Li

**Affiliations:** 1 Department of Emergency, Renmin Hospital of Wuhan University, Wuhan, Hubei Province China; 2 Department of Cardiology, Renmin Hospital of Wuhan University, Wuhan, Hubei Province China

**Keywords:** Septic cardiomyopathy, miR-31-5p, BAP1, Ferroptosis, Deubiquitination.

## Abstract

Septic cardiomyopathy is one of the most severe and common complications in patients with sepsis and poses a great threat to their prognosis. However, the potential mechanisms and effective therapeutic drugs need to be explored. The control of cardiac cell death by miRNAs has emerged as a prominent area of scientific interest in the diagnosis and treatment of heart disorders in recent times. In the present investigation, we discovered that overexpression of miR-31-5p prevented LPS-induced damage to H9C2 cells and that miR-31-5p could inhibit BAP1 production by binding to its 3’-UTR. BRCA1-Associated Protein 1 (BAP1) is a ubiquitin carboxy-terminal hydrolase. BAP1 upregulation blocked effect of miR-31-5p on H9C2 cell injury. Moreover, BAP1 inhibited the expression of solute carrier family 7 member 11 (SLC7A11) by deubiquitinating histone 2 A (H2Aub) on the promoter of SLC7A11. Furthermore, overexpression of miR-31-5p and downregulation of BAP1 inhibited SLC7A11 mediated ferroptosis. In addition, the downregulation of SLC7A11 reversed the inhibitory effect of miR-31-5p on the expression of myocardial injury and inflammatory factors, and cell apoptosis was reversed. In conclusion, these results indicate that miR-31-5p alleviates malignant development of LPS-induced H9C2 cell injury by targeting BAP1 and regulating SLC7A11 deubiquitination-mediated ferroptosis, which confirmed the protective effect of miR-31-5p on H9C2 cell injury and revealed potential mechanisms that may provide new targets for treatment of septic cardiomyopathy.

## Introduction

Sepsis is a systemic inflammatory response syndrome caused by an imbalance in the body’s response to infection owing to the invasion of pathogenic microorganisms. This reaction leads to organ dysfunction or even failure, particularly in the heart. Septic cardiomyopathy (SCM) is a common complication in patients with sepsis and is closely associated with a high incidence and mortality rate [1]. Therefore, it is imperative to identify effective targets and treatment strategies for SCM. SCM manifests in various ways, including left and right ventricular systolic and diastolic dysfunction, myocardial injury, insufficient cardiac output, and oxygen supply [2]. Elucidating the pathogenesis of SCM has become a hot topic in recent years. However, although significant progress has been made in research on SCM so far, the reported mechanisms include inflammation dysregulation caused by sepsis, mitochondrial dysfunction, energy metabolism disorders, and cellular abnormalities. However, the specific pathogenesis of SCM is still unclear [3].

Ferroptosis is a new form of programmed cell death that is caused by iron overload and lipid peroxidation. Ferroptosis occurs when the amount of iron-dependent reactive oxygen species (ROS) exceeds the iron resistance of the cells. Subsequently, peroxyphospholipid polyunsaturated fatty acids (PL-PUFA-OOH) accumulate on the cell membrane and induce membrane rupture and cell death [4]. Studies have shown that sepsis can cause an increase in iron transport and uptake and a decrease in iron output, leading to an imbalance in iron metabolism. This imbalance can increase the ROS content and disrupt the glutathione (GSH)-dependent antioxidant system, causing serious myocardial damage [5]. This indicates that inhibition of ferroptosis may help alleviate cardiac dysfunction in patients with sepsis and improve their prognosis. Recently, SLC7A11 was identified as a new target for ferroptosis in SCM [6]. SLC7A11 (member 11 of solute carrier family 7) is a multi-channel transmembrane protein responsible for the reverse transport of cysteine/glutamate. Its mechanism of action involves participation in the extracellular uptake of cystine and release of glutamate, thereby promoting the synthesis of GSH and maintaining its dependent antioxidant system [7]. As an effective anti-ferroptosis target, SLC7A11 has been shown to play a role in cancer [8], neurological diseases [9], and vascular calcification [10]. However, the role of SLC7A11 mediated ferroptosis in SCM has not been elucidated.

Histone ubiquitination and deubiquitination are the mechanisms underlying the epigenetic regulation of genes based on histone modification. The dynamic transition between ubiquitination and deubiquitination is closely related to various cellular functions [11]. Numerous studies have reported that after ubiquitination, the stability and expression of SLC7A11 are significantly reduced, leading to increased sensitivity to ferroptosis [12, 13]. Interestingly, Zhang et al. recently found that the tumor suppressor BRCA1 related protein 1 (BAP1) inhibited the transcriptional expression of SLC7A11 by deubiquitination of the ubiquitin histone H2A (H2Aub) on the SLC7A11 promoter, Histone ubiquitination is a type of post-translational modification of histones. Histone H2A is the first histone identified as ubiquitinated and H2Aub is a histone modification typically associated with transcriptional inhibition, leading to the inhibition of cysteine uptake and ferroptosis [14]. Therefore, to further explore the role of BAP1 mediated SLC7A11 deubiquitination in SCM, we predicted that miR-31-5p is an upstream target gene of BAP1 by using the GTRD database (Gene Tranion Regulation Database, http://gtrd.biouml.org/). MicroRNAs are non-coding RNAs–20–23 nucleotides in length, that play a vital role in various cardiac functions [15]. Given that miR-31-5p has been shown to reduces DOX-induced cardiac toxicity [16], we investigated the role of the targeted relationship between miR-31-5p and BAP1 in SCM.

Against this background, it is clear that the regulation of SLC7A11 deubiquitination by BAP1 may have an impact on ferroptosis in SCM. Therefore, in the present study, we investigated the regulatory effect of miR-31-5p on BAP1, and whether miR-31-5p/BAP1 inhibitd the malignant development of SCM by regulating ferroptosis. We aim to further reveal the mechanism of occurrence and development of SCM, and provide new targeted molecules for the treatment of SCM.

## Materials and methods

### Cell culture

H9C2 cells were selected for use to study the lesions of septic cardiomyopathy [17]. H9C2 cells were obtained from Procell Life Science & Technology Co.Ltd. (Procell, China). Cells were cultured in complete medium containing 10% FBS (Excell Bio, China), 90% high glucose DMEM (Gibco, China), and 1% penicillin G-streptomycin double antibody (MACKLIN, China) at 37℃ in a 5% CO_2_ atmosphere. When the cell density in the cell culture reached over 80%, the cells were digested with 1 mL 0.25% trypsin (Gibco, China) for 1 min. After the cells floated and became round, 5 mL of complete culture medium was added to terminate digestion, and the cells were passaged at a 1:3 ratio. H9C2 cells were treated with 500 µg/L lipopolysaccharide (LPS) (Thermo Fisher Scientific, USA) for 48 h to establish an in vitro model of septic cardiomyopathy.

### Cell transfection

H9C2 cells were seeded at a density of approximately 1 × 10^6^ cells per well in a 6-well plate for cultivation. For miR-31-5p, the mimic, inhibitor, and NC transfection systems were constructed and transfected into the cells according to the manufacturer’s instructions. For BAP1 and SLC7A11, 250 µL of each plasmid vector, including OE-BAP1, si-BAP1, and si-SLC7A11 (GenePharma, China), were added to 250 µL diluted LipofectamineTM^3000^ reagent (Invitrogen, USA) and incubated to form the carrier-lipid complex, which was then transfected into the cells.

### CCK-8 assay

Cell proliferation was detected using a CCK-8 Kit (Bioss, China). Logarithmically growing cells were collected. After counting, the cell suspension concentration was adjusted, and the cells were inoculated into a 96-well plate at a density of 2 × 10^3^ cells/well. According to the manufacturer’s instructions, 10 µL CCK-8 solution was added, and the cells were incubated for 2 h. The absorbance at 450 nm was measured using an RT-6000 microplate reader (Rayto, China). Cell proliferation was expressed as the cell survival rate. The calculation formula was as follows: cell survival rate (%) = (OD value (sample) - OD value (PBS)) / (OD value (control group) - OD value (PBS)) × 100%.

### Real-time PCR analysis

Total RNA was extracted from cells cultured in a six well plate was extracted using TRIzol reagent (Vazyme, China), and purified RNA was obtained after chloroform phase separation, isopropanol precipitation, and 75% ethanol washing. RNA concentration and purity were detected using Nano 600 (China), and the RNA with a A260/280 ratio between 1.9 and 2.1 were conducted in the followings. Then, A reverse transcription system was prepared that reacted at 37℃ for 15 min, 85℃ for 5 s to obtain cDNA. Next, a real-time quantitative PCR reaction system was prepared and the results were detected using CFX96 Touch 1,855,195 Real time Fluorescence Quantitative PCR Instrument (Bio-Rad, USA) according to the following procedure: pre-denaturation at 95℃ for 10 min, denaturation at 95℃ for 15 s, annealing at 58℃ for 30 s, extension at 72℃ for 30 s, and cycle count of 40; Melting curve: 95℃ for 15 s, 60℃ for 60 s, 95℃ for 15 s. Relative primer (Shanghai Sangon Co., Ltd.) sequences were as Table 1. The results were calculated using 2^−∆∆Ct^ and corrected with GAPDH as the internal reference gene.

**Table 1:** The primer sequence of Real-time PCR

Primers	NCBI ID	Sequence (5’→3’)	
miR-31-5p	NR_029747	Forward	CGAGGCAAGATGCTGGCA
(66 bp)		Reverse	AGTGCAGGGTCCGAGGTATT
BAP1	NM_001107292	Forward	ACGTTGGTGGATGATACATCTG
(157 bp)		Reverse	AGGTGGTGCGTATACGTGG
TNF-α	NM_012675	Forward	CAACAAGGAGGAGAAGTTCC
(255 bp)		Reverse	AAGAGAACCTGGGAGTAGATAAGG
IL-1β	NM_031512	Forward	CTCCATGAGCTTTGTACAAGG
(222 bp)		Reverse	TGTGCAGACTCAAACTCCAC
IL-6	NM_012589	Forward	TGAACAGCGATGATGCACTG
(351 bp)		Reverse	AGATGAGTTGGATGGTCTTGG
SLC7A11	NM_001107673	Forward	TCTCCAGGTTATACGCACATTC
(255 bp)		Reverse	CCTTGAAAGGACGATGCATATC
TFR1	NM_022712	Forward	CCCATGACATTGAGTTGACC
(340 bp)		Reverse	TTCTCCACTAAAGCTGAGAGG
IREB2	NM_022863	Forward	AGTGCTGCTGCTAAGTACTTG
(320 bp)		Reverse	AAAACAGCCTTTACACCCAGC
FTH	NM_012848	Forward	GAGAAACTGATGAAGCTGCAG
(236 bp)		Reverse	ATGGATTTCACCTGCTCATTCAGG
GAPDH	NM_017008	Forward	TTCCTACCCCCAATGTATCC
(167 bp)		Reverse	TTAGTTGCTGTTGAAGTCACAGG

### Western blot

The expression levels of BAP1, SLC7A11, GPX4, ASCL4, TNF-α, IL-1β and IL-6 were detected using western blot analysis. Briefly, total protein was extracted using RIPA lysis buffer (Beyotime, China), and a BCA kit (NCM Biotech, China) was used to determine protein concentration. The loading volume was determined to be 20 µg as the loading amount. SDS-PAGE gel electrophoresis was performed to separate the proteins, which were then transferred to a PVDF membrane (Millipore, USA). Next, After blocking with 5% skim milk powder (BD, USA), the membrane was incubated with BAP1, SLC7A11, GPX4, ASCL4, TNF-α, IL-1β and IL-6 and β-actin antibodies (Proteintech, China) overnight at 4℃. The corresponding secondary antibody (Bioss) was incubated at room temperature for 1 h on the day after washing the membrane. Finally, a chemiluminescence imaging system (Tanon, China) was used for development, and the results were analyzed using the ImageJ software for grayscale analysis.

### Flow cytometry

The apoptosis rate of H9C2 cells was detected using an Annexin V-FITC kit (Beyotime, China). After growing to cover the monolayer bottle wall, the digested cells were centrifuged and resuspended at 1000 rpm for 5 min, and then pre prepared 1X annexin V binding solution was added to prepare a cell suspension with a final concentration of 1 × 10^6^ cells/mL. Then, 5 µL Annexin V-FITC complex and 5 µL PI Solution were added to 100 µL of cell suspension and incubated at room temperature in the dark for 15 min. Next, 400 µL 1X Annexin V Binding Solution was added. Finally, fluorescence was detected within 1 h using flow cytometry, and the results were analyzed using the FlowJo software.

### CO-IP analysis

CO-IP analysis was performed to detect the ubiquitination level of SLC7A11. Total protein was extracted and quantified according to the method described in Sect. 2.6. 5 µL Protein A agarose and 5µL Protein G agarose (Beyotime, China) were added to the cell lysate and incubated at 4℃ for 1 h. Then, after centrifuging at 12,000 g, 4℃ for 1 min, 2 µg antibody, and the obtained supernatants were incubated overnight at 4℃, while using non-specific immune homologous IgG antibody as the control. The next day, the sediment was cleaned using 0.5 ml 1× wash buffer five times. The sediment was suspended in 30 µL of 1×SDS-PAGE electrophoresis sample buffer and then centrifuged for 30 s to allow the beads and liquid attached to the tube wall to reach the bottom of the tube. Then, The sample was placed at 100℃ for 5 min and centrifuged instantaneously at 14,000 g for 1 min. Finally, the collected supernatant was used for CO-IP. Western blot analysis was performed using the SLC7A11 antibody, and ubiquitination of SLC7A11 was detected using an anti-Ub antibody (Proteintech, China).

### ELISA

ELISA was performed to detect the expression levels of myocardial injury markers and inflammatory factors in cells. After treatment, the supernatant from each group of cells was collected. Standard curves were created and the expression levels of LDH and CK-MB were detected using an RT-6000 microplate reader (Rayto, China) according to the manufacturer’s instructions (Beyotime, China).

### Dual luciferase reporter assay

A dual-luciferase reporter assay was performed to observe the binding between miR-31-5p and BAP1. According to the operating instructions of the dual-luciferase reporter gene detection kit (Beyotime, China), the target gene and internal reference gene plasmids labeled with luciferase were grouped and co-transfected with H9C2 cells for 48 h. The ratio of plasmids carrying the target gene to those carrying internal references was 50:1. PmirGLO-BAP1 3-’ UTR-WT and PmirGLO-BAP1 3-UTR-mut were used as controls. Luciferase expression was measured using a JP-K6000 chemiluminescence analyzer (Jiapeng, China).

### Immunofluorescence

A MitoSOX Red fluorescent probe (Yeasen, China) was used to detect the ROS levels. After digesting the cells in the logarithmic growth phase, the cell density was adjusted to 1 × 10^6^ cells/mL in a 6 cm culture dish. Meanwhile, 13 µL DMSO was added to 50 µg MitoSOX Red Mitochondrial Superoxide Indicator to prepare a 5mM storage solution, which was then diluted to 5 µM. appropriate volume of diluted MitoSOX probe working solution was added and incubated at 37℃ for 20 min. After washing 3 times with preheated PBS, the results were observed and photographed under a fluorescence microscope. The fluorescence intensity of MitoSOX Red fluorescent probe was analyzed using ImageJ software.

### The detection of GSH and Fe^2+^

A reduced glutathione (GSH) detection kit (Zcibio, China) and iron concentration detection kit (mlbio, China) were used to detect the levels of intracellular GSH and Fe^2+^. Standard curves were created according to the manufacturer’s instructions and the results were detected using an RT-6000 microplate reader (Rayto, China).

### Statistical analysis

The experimental data are represented as the mean ± SEM, and all data were processed and statistically analyzed using GraphPad Prism 8. The comparison between the sample mean of the two groups was performed using the t-test, and the comparison between the sample mean of multiple groups was performed using the ANOVA test. *P* < 0.05, indicating that the difference was statistically significant.

## Results

### MiR-31-5p improved LPS-induced H9C2 cell injury

**Fig. 1:**
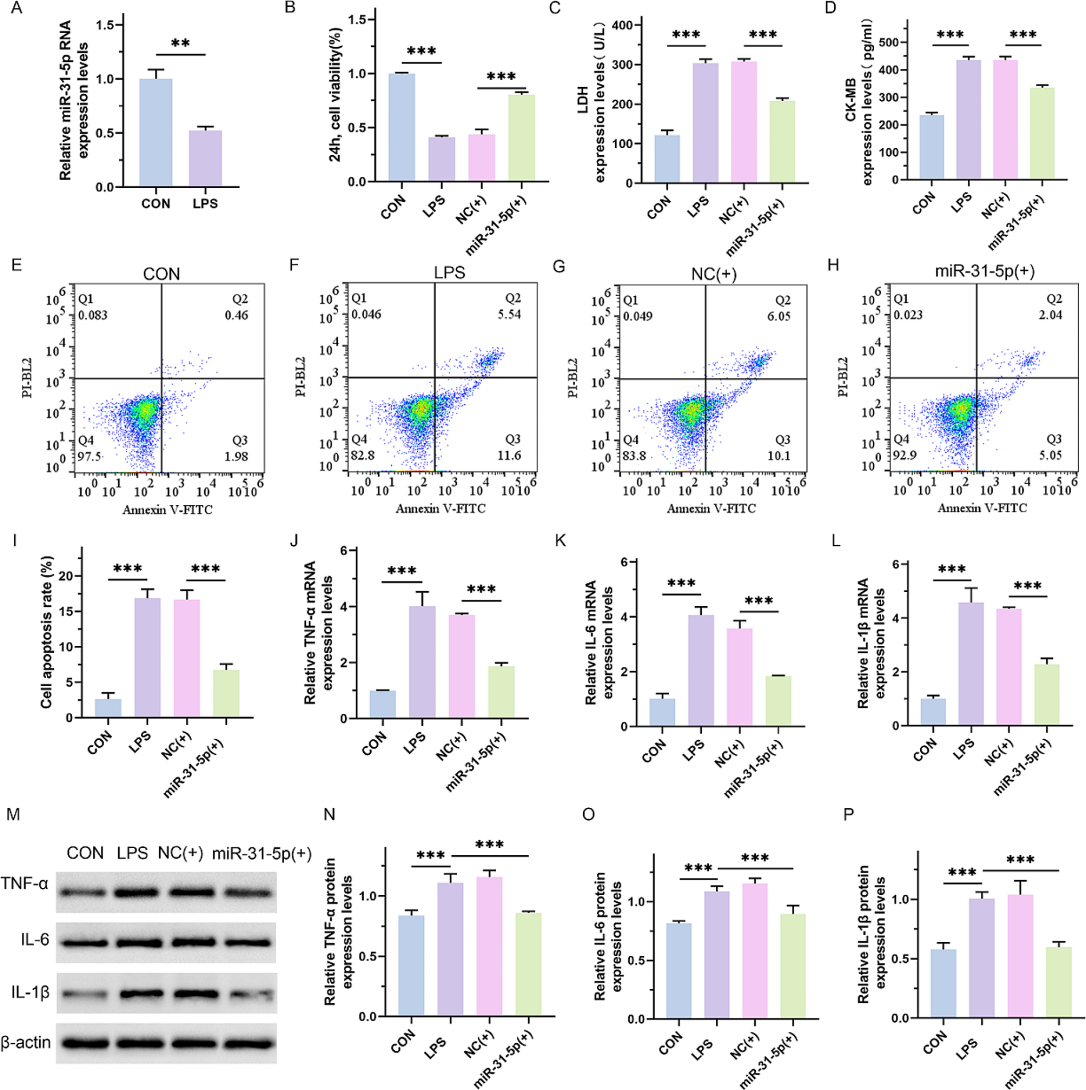
MiR-31-5p improved LPS-induced H9C2 cell injury. (**A**) Real-time PCR assay for the mRNA expression levels of miR-31-5p in H9C2 cells. The LPS group was treated by 500 µg/L LPS for 48 h. (**B**) CCK-8 assay for the proliferation ability of cells in each group. The NC(+) group was stimulated with 500 µg/L LPS and transfected with the mimics vector for 48 h; miR-31-5p(+) group was stimulated with 500 µg/L LPS and transfected with miR-31-5p mimics. (**C** and **D**) ELISA assay for the content of LDH and CK-MB. (**E**-**I**) Flow cytometry to detect apoptosis rate of H9C2 cells its quantitative analysis. (**J**-**L**) Real-time PCR assay for detecting the mRNA expression levels of TNF-α, IL-6 and IL-1β. (**M**-**P**) Western blot assay for the protein expression levels of TNF-α, IL-6 and IL-1β and the quantitative analysis. **P < 0.01 and ***P < 0.001 between indicated groups

We first exposed H9C2 cells to 500 µg/L LPS for 48 h and found that the expression of miR-31-5p was significantly decreased (Fig. 1A; *p* = 0.0038). To investigate the role of miR-31-5p in septic cardiomyopathy, we transfected miR-31-5p mimics to evaluate their impact on the cell phenotype. The CCK-8 assay showed that the proliferative ability of H9C2 cells, in which miR-31-5p was overexpressed, was obviously increased (Fig. 1B; *p* < 0.0001). ELISA showed that the levels of myocardial injury markers, including LDH and CK-MB, were decreased in the miR-31-5p overexpressing group (Fig. 1C and D; *p* < 0.0001). In addition, as revealed by the flow cytometry assay, the apoptosis rate elevated in LPS-treated H9C2 cells was reduced by miR-31-5p (Fig. 1E and I; *p* < 0.0001). In addition, the expression of inflammatory factors, including TNF-α, IL-6, and IL-1β, was decreased in the miR-31-5p overexpressing group (Fig. 1J and P; *p* < 0.0001). These results indicate that miR-31-5p inhibited the functional damage of H9C2 cells induced by LPS.

### MiR-31-5p down-regulated the expression of BAP1 in septic cardiomyopathy

**Fig. 2:**
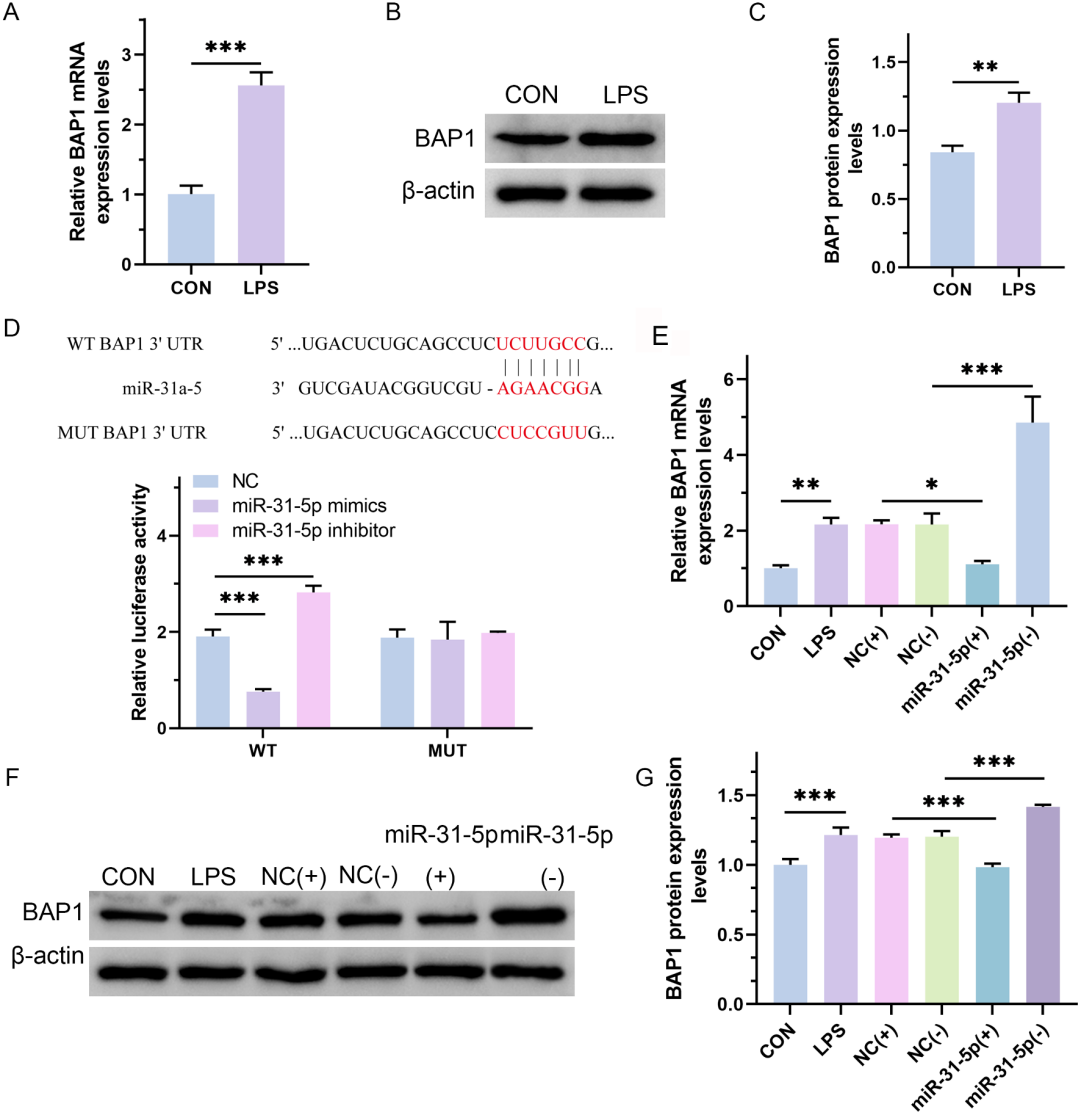
MiR-31-5p down-regulated the expression of BAP1 in septic cardiomyopathy. (**A**) Real-time PCR assay of the mRNA expression levels of BAP1. (**B** and **C**) Western blot assay for the protein expression levels of BAP1 and its quantitative analysis. (**D**) Dual-luciferase reporter assay to detect the binding site between miR-31-5p and BAP1. WT represents the wild-type sequence of BAP1, and MUT represents the mutated sequence of BAP1. (**E**-**G**) Real-time PCR and western blot assays for the mRNA and protein expression levels of BAP1 in each group. The NC(+) group was stimulated with 500 µg/L LPS and transfected with the mimic vector, NC(-) group was stimulated with 500 µg/L LPS and transfected with the inhibitor vector, miR-31-5p(+) group was stimulated with 500 µg/L LPS and transfected with miR-31-5p mimics, and miR-31-5p(-) group was stimulated with 500 µg/L LPS and transfected with miR-31-5p inhibitor. *P < 0.05, **P < 0.01 and ***P < 0.001 between indicated groups

We found that both the mRNA (Fig. 2A; *p* = 0.0006) and protein (Fig. 2B and C; *p* = 0.0036) expression of BAP1 were significantly increased in LPS-treated H9C2 cells. Based on our prediction in the database that BAP1 is a potential target of miR-31-5p, we detected the interaction relationship and binding sites between miR-31-5p and BAP1. The dual-luciferase reporter assay showed that the miR-31-5p mimic inhibited the expression of WT-BAP1 and reduced luciferase activity, indicating that BAP1 was the target of miR-31-5p (Fig. 2D; NC vs. miR-31-5p mimics, *p* < 0.0001; NC vs. miR-31-5p inhibitor, *p* = 0.0002). To confirm this, miR-31-5p mimics and inhibitor were transfected into LPS-treated H9C2 cells to observe the impact on the expression of BAP1. Real-time PCR (Fig. 2E; CON vs. LPS, *p* = 0.0083; NC(+) vs. miR-31-5p(+), *p* = 0.0152; NC(-) vs. miR-31-5p(-), *p* < 0.0001) and western blot assay (Fig. 2F and G; CON vs. LPS, *p* < 0.0001; NC(+) vs. miR-31-5p(+), *p* = 0.001; NC(-) vs. miR-31-5p(-), *p* < 0.0001) showed that the expression of BAP1 was reduced in the overexpressed miR-31-5p group and elevated in the low-expression miR-31-5p group. These results suggest that miR-31-5p inhibits LPS-induced increase in BAP1 elevated by LPS.

### Up-regulation of BAP1 reversed the protective effect of mir-31-5p in LPS-treated H9C2 cells

**Fig. 3:**
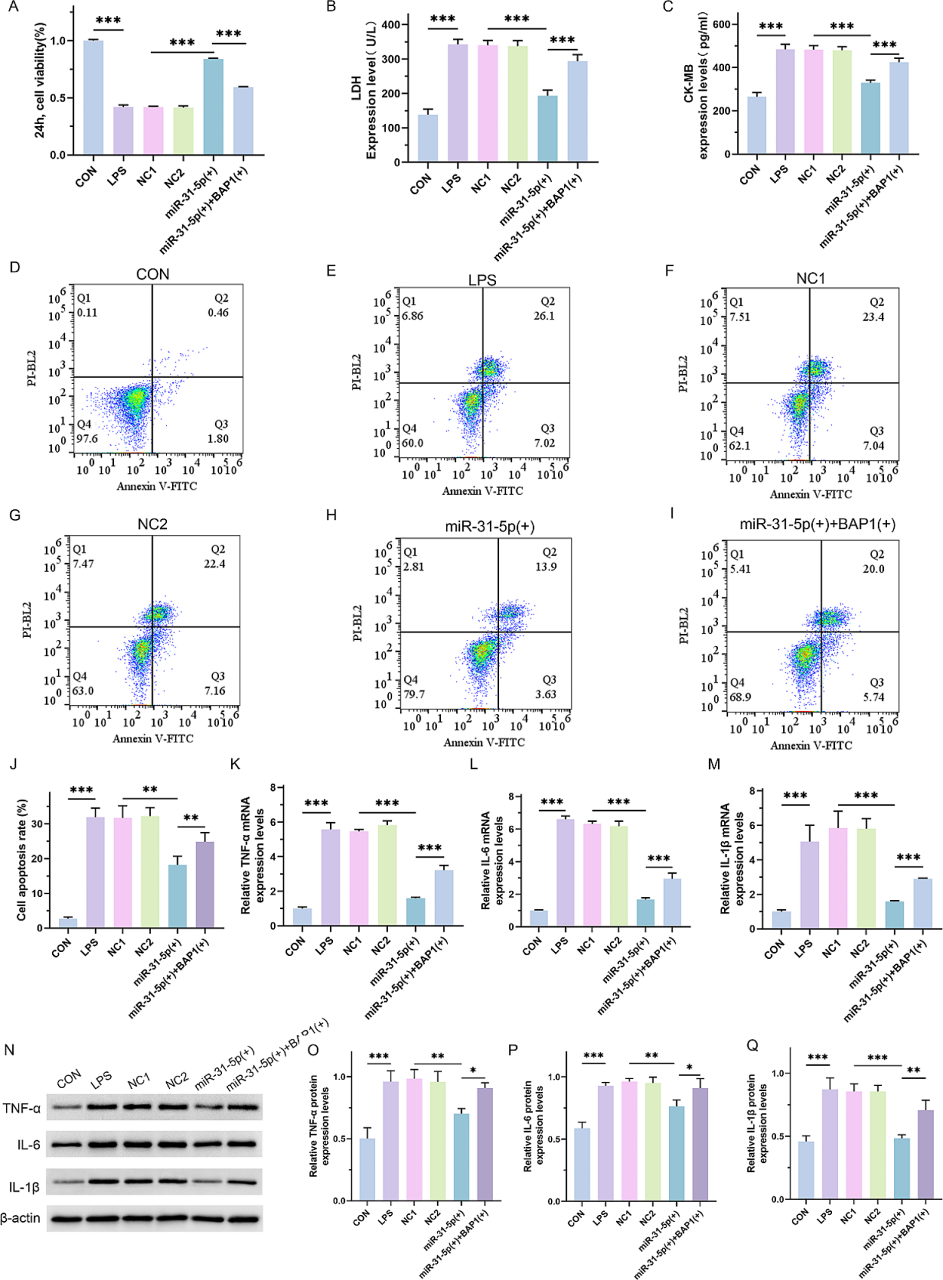
The Effect of BAP1 upregulation on the protective effect of miR-31-5p in LPS-treated H9C2 cells. (**A**) CCK-8 assay of the proliferative ability of cells in each group. The NC1 group was stimulated with 500 µg/L LPS and transfected with the mimic vector, NC2 group was stimulated with 500 µg/L LPS and transfected with lentiviral vector, miR-31-5p(+) group was stimulated with 500 µg/L LPS and transfected with miR-31-5p mimics, and miR-31-5p(+) + BAP1(+) group was stimulated with 500 µg/L LPS and transfected with miR-31-5p mimics and BAP1 lentivirus vector plasmid. (**B** and **C**) ELISA assay for LDH and CK-MB content. (**D**-**J**) Flow cytometry to detect the apoptosis rate of H9C2 cells and quantitative analysis. (**K**-**M**) Real-time PCR assay for detecting the mRNA expression levels of TNF-α, IL-6, and IL-1β. (**N**-**Q**) Western blot assay for the protein expression levels of TNF-α, IL-6 and IL-1β and the quantitative analysis. *P < 0.05, **P < 0.01 and ***P < 0.001 between indicated groups

To clarify whether miR-31-5p improved septic cardiomyopathy by regulating BAP1, the BAP1 lentivirus vector plasmid was transfected into the miR-31-5p overexpressing group to observe its effect on miR-31-5p inhibiting septic cardiomyopathy. As expected, the results showed that compared with the miR-31-5p overexpression group, BAP1 significantly weakened the viability of H9C2 cells (Fig. 3A; *p* < 0.0001) and increased the levels of LDH and CK-MB (Fig. 3B and C; *p* < 0.0001). Moreover, in the BAP1 upregulated group, both the apoptosis rate of H9C2 cells (Fig. 3D and J; CON vs. LPS, *p* < 0.0001; NC1 vs. miR-31-5p(+), *p* = 0.0016; miR-31-5p(+) vs. miR-31-5p(+) + BAP1(+), *p* = 0.0087) elevated. Real-time PCR (Fig. 3K and M; *p* < 0.0001) and western blot assay (Fig. 3N, Q and O, CON vs. LPS, *p* < 0.001; NC1 vs. miR-31-5p(+), *p* = 0.004; miR-31-5p(+) vs. miR-31-5p(+) + BAP1(+), *p* = 0.04; Fig. 3P, CON vs. LPS, *p* < 0.001; NC1 vs. miR-31-5p(+), *p* = 0.003; miR-31-5p(+) vs. miR-31-5p(+) + BAP1(+), *p* = 0.03; Fig. 3Q, CON vs. LPS, *p* < 0.001; NC1 vs. miR-31-5p(+), *p* < 0.001; miR-31-5p(+) vs. miR-31-5p(+) + BAP1(+), *p* = 0.009) showed that the expression levels of inflammatory factors were notably elevated. Collectively, after BAP1 overexpression, the inhibitory effect of miR-31-5p on the malignant transformation of LPS-induced H9C2 was reversed. These results suggest that miR-31-5p inhibits LPS-induced H9C2 cell injury by downregulating BAP1 expression.

### BAP1 inhibited the expression of SLC7A11 through deubiquitination

**Fig. 4:**
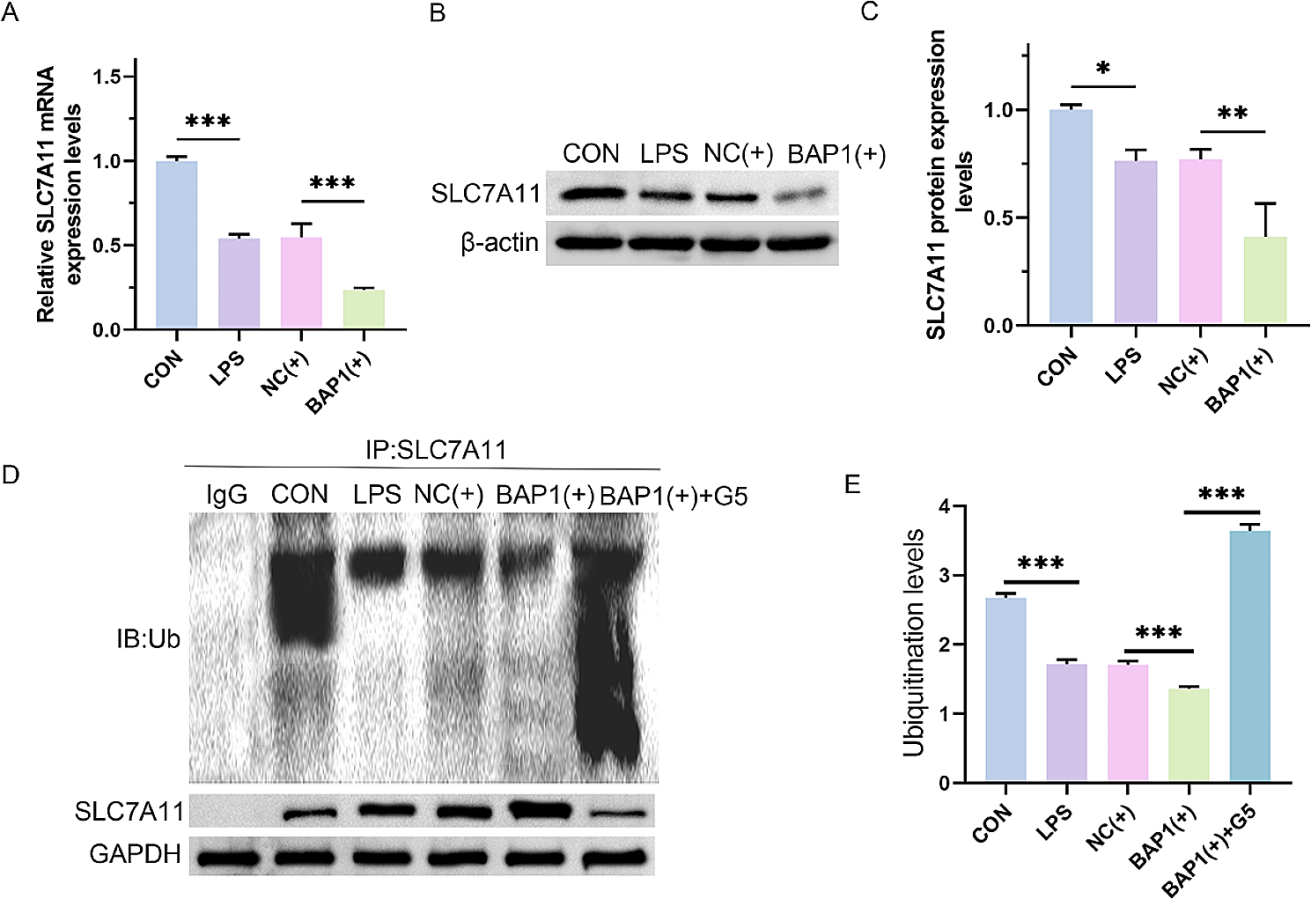
BAP1 inhibits the expression of SLC7A11 through deubiquitination. (**A**-**C**) Real-time PCR and western blot assays for the mRNA and protein expression levels of SLC7A11 in H9C2 cells. The NC(+) group was stimulated with 500 µg/L LPS and transfected with a lentiviral vector and the BAP1(+) group was stimulated with 500 µg/L LPS and transfected with the BAP1 lentivirus vector plasmid. (**D**) CO-IP assay to detect the ubiquitination levels of SLC7A11. The BAP1(+) + G5 group was stimulated with 500 µg/L LPS and treated with BAP1 lentiviral vector and 1 µM G5. (**E**) Quantitative analysis of CO-IP assay. *P < 0.05, **P < 0.01 and ***P < 0.001 between indicated groups

Next, we determined the regulatory effects of BAP1 on the expression of SLC7A11. Real-time PCR and western blotting assays showed that as a classic anti-ferroptosis protein, SLC7A11 was down-regulated in LPS-stimulated H9C2 cells. Moreover, after overexpression of BAP1, the mRNA (Fig. 4A; CON vs. LPS, *p* < 0.0001; NC(+) vs. BAP1(+), *p* = 0.0001) and protein expression level (Fig. 4B and C; CON vs. LPS, *p* = 0.0391; NC(+) vs. BAP1(+), *p* = 0.0041) of SLC7A11 were both further decreased. To determine whether BAP1 induced the deubiquitination of SLC7A11, the deubiquitination enzyme inhibitor G5 was added to examine its effect on the ubiquitination level of SLC7A11. As illustrated by the CO-IP assay, overexpression of BAP1 reduced the ubiquitination level of SLC7A11, whereas the addition of G5 increased the ubiquitination level (Fig. 4D and E; CON vs. LPS, *p* < 0.0001; NC(+) vs. BAP1(+), *p* = 0.0002; BAP1(+) vs. BAP1(+) + G5, *p* < 0.0001). These findings imply that BAP1 induces the deubiquitination of SLC7A11, thereby inhibiting its expression.

### BAP1 induced ferroptosis by inhibiting SLC7A11

**Fig. 5:**
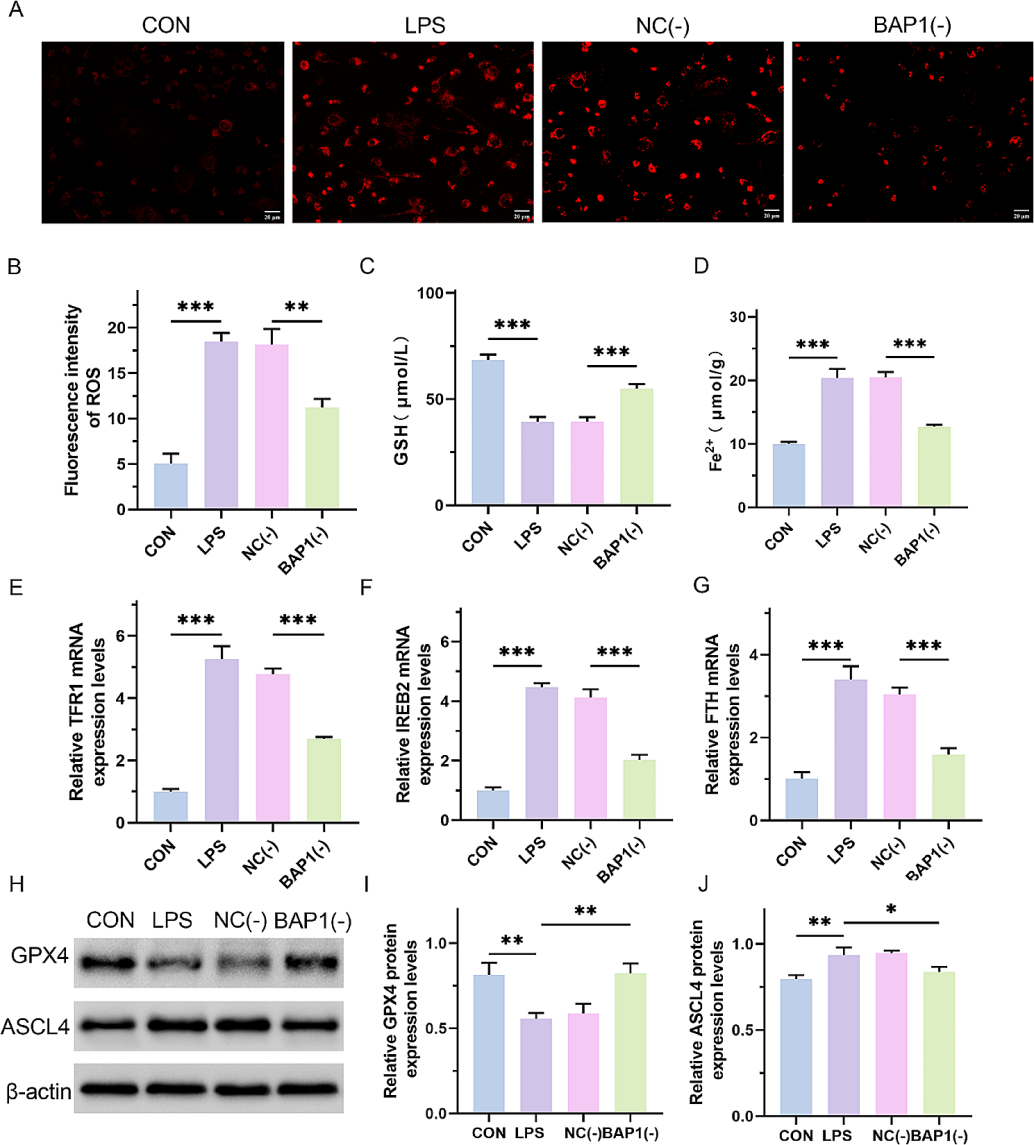
BAP1 induces ferroptosis by inhibiting SLC7A11. (**A** and **B**) Immunofluorescence staining using the MitoSOX Red fluorescence probe for detecting intracellular ROS content and quantitative analysis. Scale bar, 20 μm. The NC(-) group was stimulated with 500 µg/L LPS and transfected with the siRNA vector, and the BAP1(-) group was stimulated with 500 µg/L LPS and transfected with the si-BAP1 plasmid. (**C** and **D**) GSH and Fe^2+^ content in each group. (**E**-**G**) Real-time PCR assay to detect the mRNA expression levels of TFR1, IREB2, and FTH. (**H**-**J**) Western blot assay for the protein expression levels of GPX4 and ASCL4 and the quantitative analysis. *P < 0.05, **P < 0.01 and ***P < 0.001 between indicated groups

To further explore the role of BAP1 mediated SLC7A11 deubiquitination in septic cardiomyopathy, a si-BAP1 plasmid was transfected to observe its effect on ferroptosis. An immunofluorescence assay based on the MitoSOX Red fluorescence probe was performed to detect the intracellular ROS content. inhibiting expression of BAP1, the ROS content was significantly decreased (Fig. 5A and B; CON vs. LPS, *p* = 0.0003; NC(-) vs. BAP1(-), *p* = 0.0024). In addition, in the BAP1 inhibition group, the GSH content increased (Fig. 5C; *p* < 0.0001), and the Fe^2+^ content decreased (Fig. 5D; *p* < 0.0001). Meanwhile, the expression levels of transferrin receptor 1 (TFRl), iron responsive element binding protein 2 (IREB2), and ferritin heavy chain (FTH), which are related to iron metabolism, also decreased (Fig. 5E and G; *p* < 0.0001). Moreover, as revealed by western blot assay, knockdown of BAP1 also upregulated the expression of GPX4 reduced by LPS (Fig. 5H and I; CON vs. LPS, *p* = 0.002; NC(-) vs. BAP1(-), *p* = 0.004) and downregulated the expression of ASCL4 elevated by LPS (Fig. 5H and J; CON vs. LPS, *p* = 0.002; NC(-) vs. BAP1(-), *p* = 0.01), which were the marker proteins of ferroptosis. Our results indicated that BAP1 induces ferroptosis in LPS-treated H9C2 cells.

### MiR-31-5p inhibited ferroptosis in H9C2 cells

**Fig. 6:**
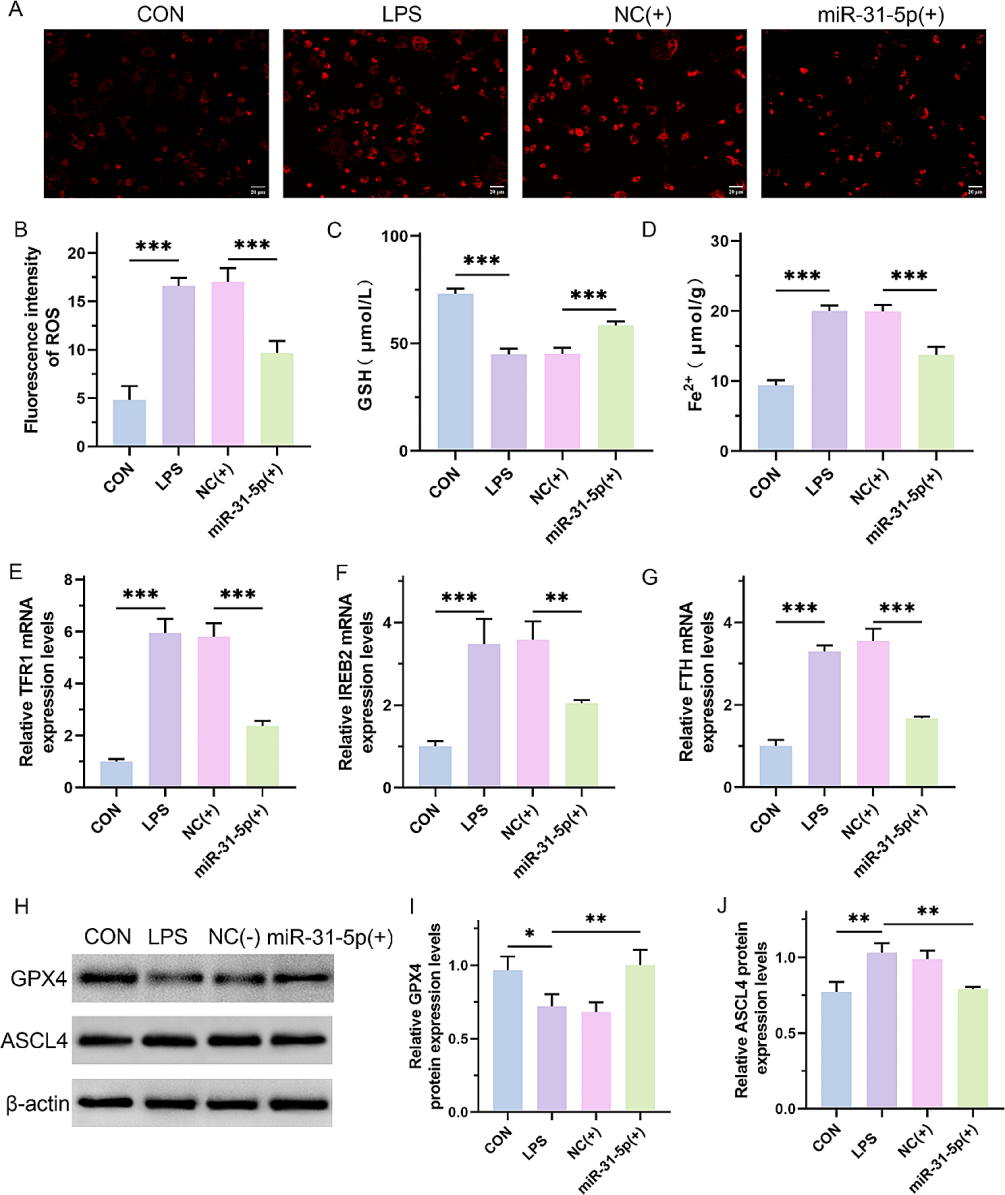
MiR-31-5p inhibited ferroptosis in H9C2 cells. (**A** and **B**) Immunofluorescence staining using the MitoSOX Red fluorescence probe for detecting intracellular ROS content and quantitative analysis. Scale bar, 20 μm. The NC(+) group was stimulated with 500 µg/L LPS and transfected with the mimic vector, and the miR-31-5p(+) group was stimulated with 500 µg/L LPS and transfected with miR-31-5p mimics. (**C** and **D**) GSH and Fe^2+^ content in each group. (**E**-**G**) Real-time PCR assay to detect the mRNA expression levels of TFR1, IREB2, and FTH. (**H**-**J**) Western blot assay for the protein expression levels of GPX4 and ASCL4 and the quantitative analysis. *P < 0.05, **P < 0.01 and ***P < 0.001 between indicated groups

As shown above, BAP1 promotes the deubiquitination of SLC7A11, leading to ferroptosis in H9C2 cells. Thus, we confirmed the role of ferroptosis in the miR-31-5p mediated alleviation of H9C2 cell injury. The MitoSOX Red fluorescence probe showed that miR-31-5p reduced the ROS levels in H9C2 cells (Fig. 6A and B; *p* < 0.0001). Furthermore, miR-31-5p also increased GSH content and reduced Fe^2+^ content (Fig. 6C and D; *p* < 0.0001). In addition, miR-31-5p decreased the expression of TFRl (Fig. 6E; *p* < 0.0001), IREB2 (Fig. 6F; CON vs. LPS, *p* = 0.0002; NC(+) vs. miR-31-5p(+), *p* = 0.0051), and FTH (Fig. 6G; *p* < 0.0001). Besides, the expression of GPX4 was notably increased with overexpression of miR-31-5p (Fig. 6H and I; CON vs. LPS, *p* = 0.04; NC(+) vs. miR-31-5p(+), *p* = 0.009) and the expression of ASCL4 was decreased in miR-31-5p overexpression group (Fig. 6H and J; CON vs. LPS, *p* = 0.002; NC(+) vs. miR-31-5p(+), *p* = 0.009). In summary, miR-31-5p prevents LPS treatment-induced ferroptosis in H9C2 cells.

### The effect of inhibiting SLC7A11 expression on mir-31-5p improving H9C2 cell injury

**Fig. 7:**
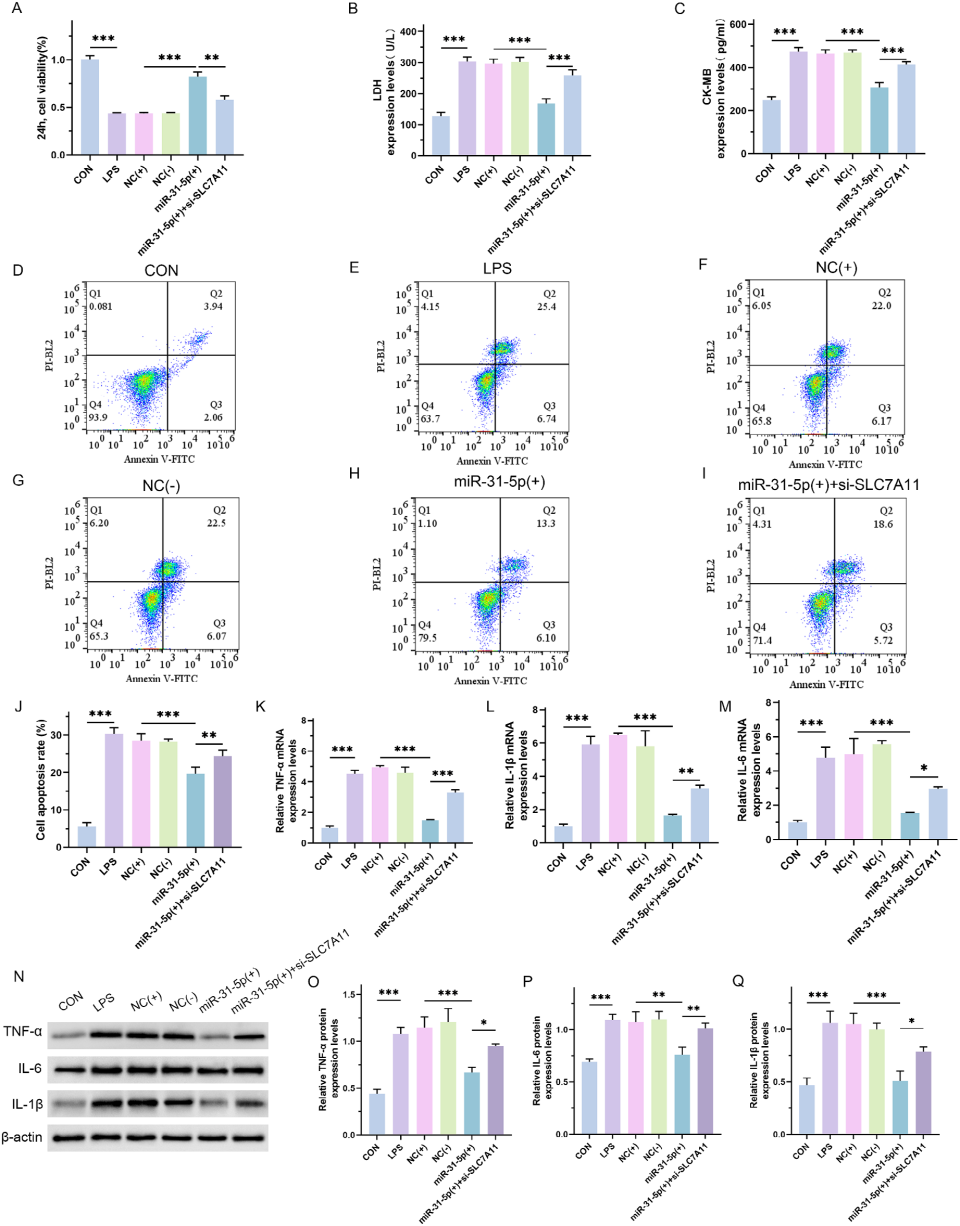
Effect of inhibiting SLC7A11 expression on miR-31-5p improves H9C2 cell injury. (**A**) CCK-8 assay of the proliferative ability of cells in each group. The NC(+) group was stimulated with 500 µg/L LPS and transfected with the mimic vector, NC(-) group was stimulated with 500 µg/L LPS and transfected with siRNA vector, miR-31-5p(+) group was stimulated with 500 µg/L LPS and transfected with miR-31-5p mimics, and miR-31-5p(+) + si-SLC7A11 was stimulated with 500 µg/L LPS and transfected with miR-31-5p mimics and si-SLC7A11 plasmid. (**B** and **C**) ELISA assay for LDH and CK-MB content. (**D**-**J**) Flow cytometry to detect the apoptosis rate of H9C2 cells and quantitative analysis. (**K**-**M**) Real-time PCR assay for detecting the mRNA expression levels of TNF-α, IL-6, and IL-1β. (**N**-**Q**) Western blot assay for the protein expression levels of TNF-α, IL-6 and IL-1β and the quantitative analysis. *P < 0.05, **P < 0.01 and ***P < 0.001 between indicated groups

To verify the involvement of SCL7A11 in the resistance of miR-31-5p/BAP1 to septic cardiomyopathy, we then used the construction of si-SLC7A11 plasmid to transfect H9C2 cells. It was showed that compared with the miR-31-5p over-expressed group, knocking down the expression of SLC7A11 resulted in a decrease in cell viability, an increase in the content of myocardial injury markers (Fig. 7A and C; *p* < 0.0001), and an increase in cell apoptosis rate (Fig. 7D and J; CON vs. LPS, *p* < 0.0001; NC(+) vs. miR-31-5p(+), *p* < 0.0001; miR-31-5p(+) vs. miR-31-5p(+) + si-SLC7A11, *p* = 0.0069). In addition, Real-time PCR (Fig. 7K, M and K, *p* < 0.0001; Fig. 7L, CON vs. LPS, *p* < 0.001; NC(+) vs. miR-31-5p(+), *p* < 0.0001; miR-31-5p(+) vs. miR-31-5p(+) + si-SLC7A11, *p* = 0.007) and western blot assay (Fig. 7N-3Q; Fig. 7O, CON vs. LPS, *p* < 0.001; NC(+) vs. miR-31-5p(+), *p* < 0.001; miR-31-5p(+) vs. miR-31-5p(+) + si-SLC7A11, *p* = 0.02; Fig. 7P, CON vs. LPS, *p* < 0.001; NC(+) vs. miR-31-5p(+), *p* = 0.001; miR-31-5p(+) vs. miR-31-5p(+) + si-SLC7A11, *p* = 0.007; Fig. 7Q, CON vs. LPS, *p* < 0.001; NC(+) vs. miR-31-5p(+), *p* < 0.001; miR-31-5p(+) vs. miR-31-5p(+) + si-SLC7A11, *p* = 0.01) showed that the downregulation of SLC7A11 expression also led to an increase in the expression levels of inflammatory factors reduced by miR-31-5p. Taken together, these results demonstrate that the protective effect of miR-31-5p on H9C2 cell injury is related to the upregulation of the expression of the anti-ferroptosis protein SLC7A11.

## Discussion

In the current study, miR-31-5p/BAP1 was shown to inhibit LPS-induced H9C2 cell injury. BAP1 has been shown to induce deubiquitination of H2Aub on the SLC7A11 promoter. Downregulation of SLC7A11 expression led to cell ferroptosis, and the protective effect of miR-31-5p may be related to its inhibition of BAP1 induced ferroptosis.

The functions of miRNAs in a number of disorders have garnered a lot of interest lately [18]. We discovered in this work that miR-31-5p guards against H9C2 damage brought on by LPS. Numerous studies indicate that the inflammatory response is a major factor in heart damage and even failure, and that sepsis can impair cardiac function [19]. We verified that miR-31-5p overexpression improved cell survival and reduced the expression of markers for inflammatory infiltration and myocardial damage. Based on these information, miR-31-5p is a useful target for the management of septic cardiomyopathy.

In addition to being oncogenes or tumor suppressors, miRNAs can regulate the expression of downstream oncogenes or tumor suppressors [20]. We found that BAP1 was a downstream target of miR-31-5p. The tumor inhibitor BAP1 is a ubiquitin carboxyl-terminal hydrolase with deubiquitination activity that is involved in regulating many cellular processes, such as DNA damage repair and programmed cell death [21]. BAP1 has become a research hotspot in the field of cancer because of its ability to reduce histone 2 A ubiquitination (H2Aub) on the downstream protein chromatin [22]. However, there aren’t any reports as of now about BAP1’s function in cardiac damage. Here, we report, for the first time, that septic cardiomyopathy is associated with elevated BAP1. Exogenous overexpression of miR-31-5p could inhibit the expression of BAP1 in LPS treated H9C2 cells. Most importantly, miR-31-5p’s protective impact against septic cardiomyopathy was inhibited following the up-regulation of BAP1 expression. Therefore, inhibiting BAP1 expression by overexpressing miR-31-5p may be a promising approach for treating septic cardiomyopathy.

It’s interesting to note that miR-31-5p improves the malignant progression of septic cardiomyopathy by inhibiting BAP1. According to a report by Zhang et al., BAP1 causes the deubiquitination of H2Aub, which prevents SLC7A11 from being expressed [14]. This was confirmed in the current investigation. As SLC7A11 is an anti-ferroptosis protein, several studies have demonstrated that downregulating SLC7A11 expression is harmful to myocardial damage. Ye et al. found that vascular smooth muscle cell calcification was accelerated by downregulating SLC7A11 expression [10]. Zhu et al. discovered that inhibition of SLC7A11 exacerbated doxorubicin-induced cardiac toxicity [23]. Lin et al. reported that activation of the SIRT1/pp53/SLC7A11 axis could alleviate septic cardiomyopathy [24]. These factors are related to myocardial cell ferroptosis. While the buildup of ROS is the main factor leading to disruption of the GSH-dependent antioxidant system, the inhibition of cysteine/glutamate reverse transporters is a critical mechanism initiating ferroptosis [25]. The dysfunction of the antioxidant system can lead to abnormal iron metabolism, manifested by increased expression of iron metabolism markers TFR1, IREB2, and FTH [26]. In this study, we found that overexpression of miR-31-5p or inhibition of BAP1 expression decreased the levels of intracellular ROS, Fe^2+^, and iron metabolism markers, while the GSH content was increased. Therefore, regulating the miR-31-5p/BAP1 axis may inhibit myocardial cell ferroptosis.

In summary, our study demonstrated the interaction between miR-31-5p and BAP1 and revealed the molecular mechanism by which miR-31-5p/BAP1 regulated SLC7A11 mediated ferroptosis by deubiquitination in alleviating LPS-induced H9C2 cell injury. These findings provide an important theoretical basis for targeted diagnosis and treatment of septic cardiomyopathy. However, the mechanism of the occurrence and development of septic cardiomyopathy is complex, and research at the cellular level is also limited. Our conclusion needs to be validated at the animal level, and its deeper mechanisms also require more research.

## Data Availability

The raw data used in the present study had been uploaded in the Figshare. Please check the following website: 10.6084/m9.figshare.25045805.
